# HyperSpec: Ultrafast Mass Spectra Clustering in Hyperdimensional Space

**DOI:** 10.1021/acs.jproteome.2c00612

**Published:** 2023-05-11

**Authors:** Weihong Xu, Jaeyoung Kang, Wout Bittremieux, Niema Moshiri, Tajana Rosing

**Affiliations:** † Department of Computer Science Engineering, University of California, San Diego, La Jolla, California 92093, United States; ‡ Department of Electrical and Computer Engineering, University of California, San Diego, La Jolla, California 92093, United States; § Department of Computer Science, University of Antwerp, 2020 Antwerpen, Belgium

**Keywords:** mass spectrometry, spectral clustering, peptide identification, hyperdimensional computing, runtime optimization

## Abstract

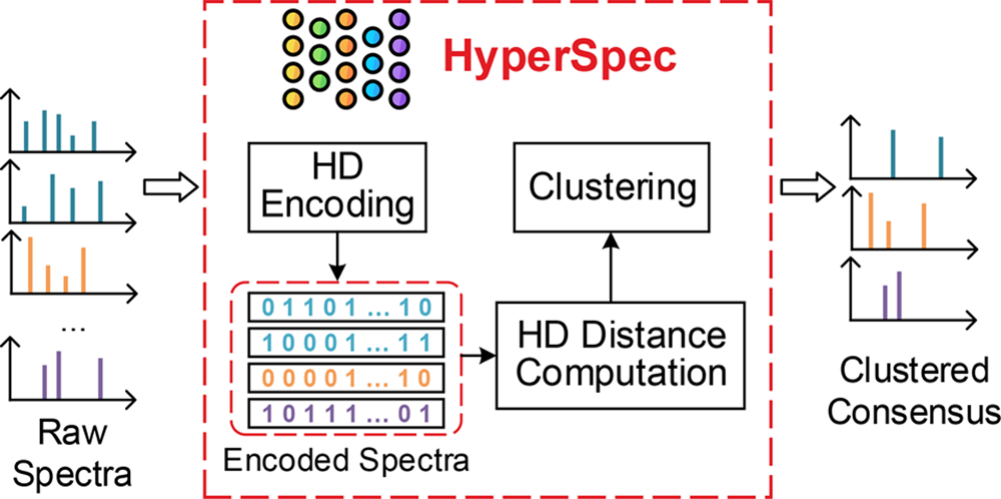

As current shotgun proteomics experiments can produce gigabytes of mass spectrometry data per hour, processing these massive data volumes has become progressively more challenging. Spectral clustering is an effective approach to speed up downstream data processing by merging highly similar spectra to minimize data redundancy. However, because state-of-the-art spectral clustering tools fail to achieve optimal runtimes, this simply moves the processing bottleneck. In this work, we present a fast spectral clustering tool, HyperSpec, based on hyperdimensional computing (HDC). HDC shows promising clustering capability while only requiring lightweight binary operations with high parallelism that can be optimized using low-level hardware architectures, making it possible to run HyperSpec on graphics processing units to achieve extremely efficient spectral clustering performance. Additionally, HyperSpec includes optimized data preprocessing modules to reduce the spectrum preprocessing time, which is a critical bottleneck during spectral clustering. Based on experiments using various mass spectrometry data sets, HyperSpec produces results with comparable clustering quality as state-of-the-art spectral clustering tools while achieving speedups by orders of magnitude, shortening the clustering runtime of over 21 million spectra from 4 h to only 24 min.

## 1 Introduction

Mass spectrometry (MS) is currently the dominant analytical technique to analyze the protein composition of biological samples and study the proteome.^1−3^ Fueled by progress in instrumentation over the previous decade, modern MS experiments can consist of millions of mass spectra and require tens to hundreds of gigabytes storage space. However, because a typical spectral identification workflow consists of exhaustively comparing each collected MS/MS spectrum against the digested protein database to find relevant peptide–spectrum matches, the generation of increasingly large data sets can become problematic as MS data analysis becomes excessively time consuming. For example, analyzing a large-scale draft human proteome data set,^1^ amounting to 25 million MS/MS spectra and 131 GB of MS data, requires several hours to days of processing time.

Spectral clustering is an effective approach to shortening the spectral identification runtime by reducing the search space.^4−9^ Prior to peptide identification, highly similar MS/MS spectra are first clustered together, and each cluster is represented by a consensus spectrum. The benefits of this approach are 3-fold. First, clustering minimizes data redundancy by grouping repeated MS/MS spectra and representing them as a single consensus spectrum. Second, rather than having to analyze all raw spectra, downstream tools only need to process a smaller number of consensus spectra. For example, Wang et al.^4^ reported using spectral clustering to reduce the runtime of subsequent peptide identification by over 50%. Third, the downstream analysis can achieve better results by operating on high-quality consensus spectra with a higher signal-to-noise ratio compared to the raw spectra.^10^

Previous spectral clustering tools have focused on optimizing clustering quality and clustering speed. For example, MS-Cluster^5^ and spectra-cluster^8^ use an iterative greedy approach to efficiently merge similar spectra. spectra-cluster has been utilized for large-scale clustering of public MS data in the PRoteomics IDEntifications (PRIDE) database^11^ to build the PRIDE-Cluster spectral libraries. MaRaCluster^6^ proposed an optimized similarity metric that relies on the rarity of fragment peaks to compare MS/MS spectra. Based on the intuition that peaks shared by only a few spectra offer more evidence than peaks shared by a large number of spectra, relative to a background frequency of fragment peaks with specific *m*/*z* values, matches of frequent fragment peaks contribute less to the spectrum similarity than matches of rare peaks. Next, MaRaCluster performs hierarchical clustering with complete linkage to group similar spectra in clusters. However, the clustering speed of MS-Cluster, spectra-cluster, and MaRaCluster can be extremely slow on large data sets as they run on CPU hardware only, which lacks massive parallelism. For example, Bittremieux et al.^9^ reported that these tools took 8–30 h to cluster a draft human proteome data set consisting of 25 million MS/MS spectra.^1^ Unfortunately, such long clustering runtime negates any potential benefits of shortened runtime from downstream applications. Additionally, as current MS data repositories contain orders of magnitude more data, with several billions of MS/MS spectra currently available, performing spectral clustering at the repository scale becomes increasingly challenging and computationally infeasible.

Several spectral clustering tools have tried to address this issue by focusing on processing speed. msCRUSH^4^ utilizes locality-sensitive hashing (LSH) to achieve fast clustering speeds by projecting similar spectra into shared LSH buckets to avoid unnecessary pairwise spectrum comparisons. Within each bucket, msCRUSH then uses a greedy spectrum merging strategy similar to MS-Cluster and spectra-cluster to cluster the spectra. falcon^9^ first converts spectra to low-dimensional vectors using a hashing strategy. It uses approximate nearest neighbor searching^12^ to construct a sparse pairwise distance matrix, which helps to shorten the required runtime. ClusterSheep^7^ further optimizes the spectral clustering runtime by offloading computations to a graphics processing unit (GPU). Compared to falcon, ClusterSheep implements function kernels on a GPU to speed up further. Unfortunately, however, despite their efficient runtimes, falcon and ClusterSheep exhibit some reduction in clustering quality compared to MaRaCluster and msCRUSH. Consequently, existing spectral clustering tools still lack the ability to yield high clustering quality within a short runtime when processing large-scale data sets.

Here, we propose HyperSpec, a GPU-accelerated spectral clustering library using brain-inspired hyperdimensional computing (HDC).^13^ Unlike previous hashing-based methods that project spectra into a low-dimensional space, HDC instead encodes spectra into binary high-dimensional vectors, called hypervectors (HVs). Compared to the low-dimensional embeddings used by msCRUSH^4^ and falcon,^9^ HVs are superior in the sense that spectra can be encoded as compact, binary vector representations with a minimal loss of information. Additionally, binary HDC operation in HyperSpec offers high data parallelism for low-level hardware architecture, which we leveraged by developing fast Python kernels tailored for exploiting GPU resources. By operating on spectra represented as HVs, HyperSpec achieves state-of-the-art clustering quality and clustering speed. Our experiments show that HyperSpec is scalable to different data set sizes and significantly accelerates spectral clustering up to 15× compared to alternative clustering tools. As an example, clustering of a large draft human proteome data set^1^ was reduced from over 4 h (using MaRaCluster) to only 24 min. Meanwhile, the peptide identification quality using clustered consensuses generated by HyperSpec is comparable with state-of-the-art tools. HyperSpec is freely available as open source on GitHub at https://github.com/wh-xu/Hyper-Spec under the BSD license.

## 2 Materials and Methods

### 2.1 Overall Flow

**Figure 1:**
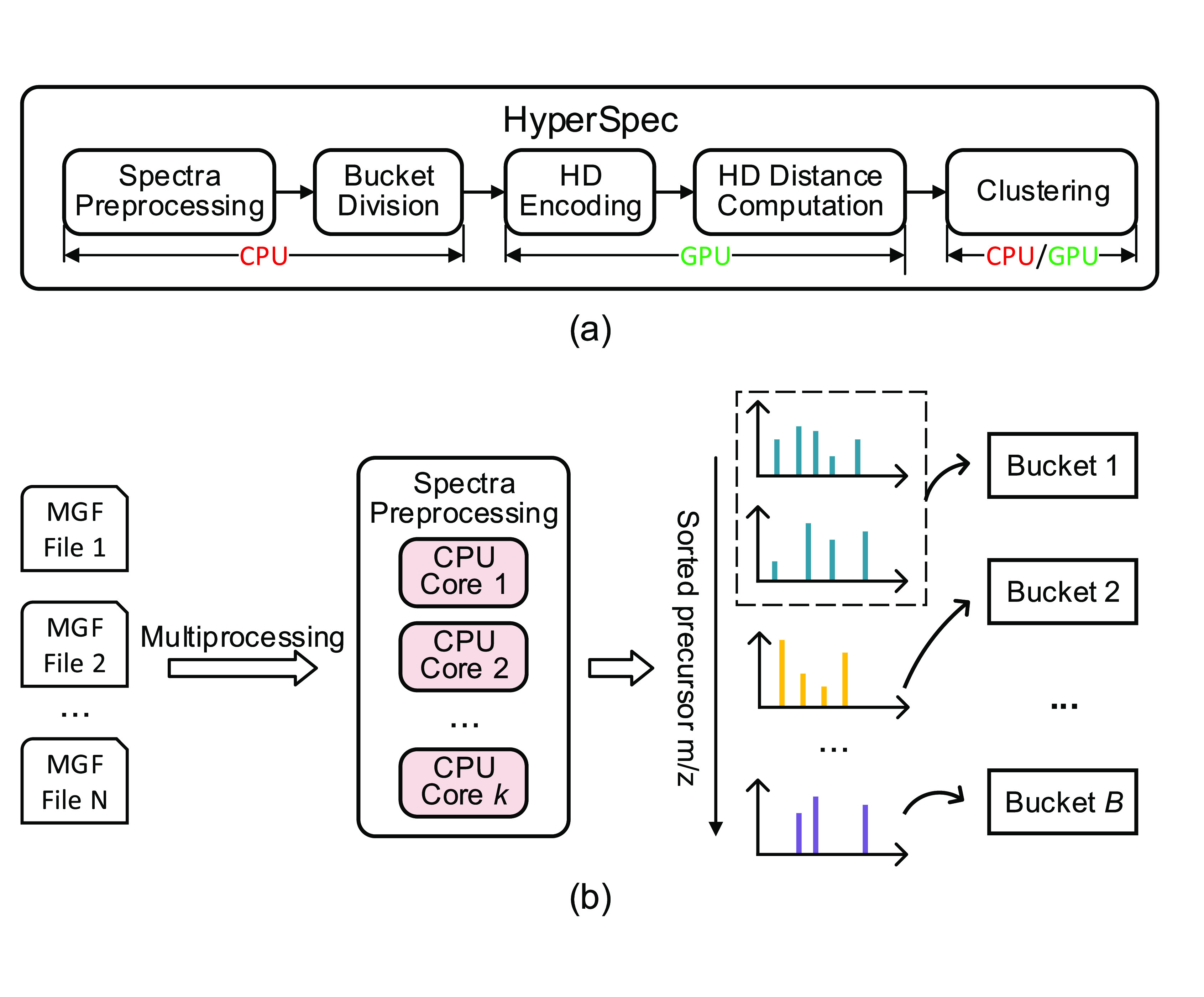
(a) Overall diagram of HyperSpec. (b) HyperSpec’s spectrum preprocessing and bucket division flow. HyperSpec’s spectra preprocessing and bucket division are optimized using multiprocessing on a CPU. HD encoding and distance computation are offloaded to a highly parallel GPU.

HyperSpec is a Python library for spectral clustering that optimally makes use of both CPU and GPU hardware resources (Figure 1a). The overall data processing flow of HyperSpec consists of five main steps, including spectrum preprocessing, bucket division, hyperdimensional (HD) encoding, HD distance computation, and clustering. The first two steps, namely, spectrum preprocessing and bucket division, are executed on a CPU, while the HD encoding and distance computation are accelerated using GPUs. This allows HyperSpec to fully utilize both CPU and GPU computing resources for optimized preprocessing and clustering speed.

### 2.2 Efficient Spectrum Preprocessing

**Figure 2:**
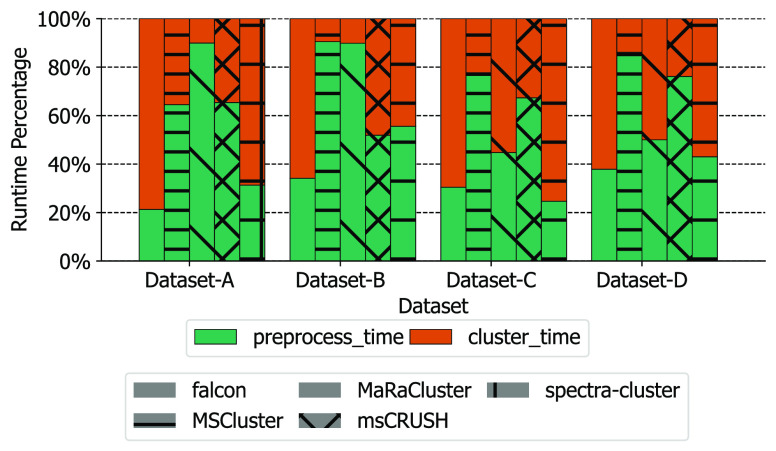
Runtime profiling for five popular spectral clustering tools (falcon,^9^ msCRUSH,^4^ MaRaCluster,^6^ spectra-cluster,^8^ and MS-Cluster^5^). The runtimes were evaluated in terms of the time required for spectrum preprocessing and the time required for spectral clustering.

Prior to spectral clustering, the raw spectra need to be loaded and preprocessed. This is one of the bottlenecks during spectral clustering, contributing 20–90% of the overall runtime for several state-of-the-art spectral clustering tools (Figure 2). There are several reasons contributing to the slow preprocessing step. (1) During preprocessing, raw spectra are loaded and parsed from the storage device, after which the parsed spectra are processed to remove noise. The parsing phase is bounded by the speed of parsing spectral data into a numerical format, since the peak information takes up over 95% of the data volume in raw files. (2) Processing the parsed spectra is computation bounded, because it requires sorting, computing, and data manipulation for high-dimensional peak vectors. (3) Another crucial factor limiting the preprocessing speed of existing clustering tools is the underutilization of storage I/O bandwidth. Specifically, modern solid-state drives (SSD) provide GB/s sequential access speeds, but most clustering tools cannot provide sufficient preprocessing speed to match the I/O bandwidth. To this end, HyperSpec optimizes spectrum preprocessing as follows.

#### 2.2.1 Multiprocessing

HyperSpec uses the commonly used Mascot Generic Format (MGF) as an input. HyperSpec utilizes multiprocessing to read each file in parallel and distribute the computation to *k* independent CPU cores (Figure 1b). Spectrum preprocessing is composed of two phases: spectrum parsing and preprocessing. We implemented an optimized spectrum data parser and a parallelized preprocessor, which are executed independently on *k* CPU cores to increase data parallelism. Supplementary Information Figure S1 shows that multiprocessing achieves sublinear speedup and effectively reduces the preprocessing time.

#### 2.2.2 Spectrum Data Parser

Rather than using standalone C++ or Python parsing,^4,5,9^ HyperSpec uses a hybrid C++–Python program, which balances Python’s convenience of code extension and C++’s performance. The low-level spectrum data parser is built using the Spirit X3 parser in Boost C++^14^ to convert MGF data to numerical arrays. After being compiled, the spectrum data parser is invoked by the high-level Python interface using multiprocessing.

#### 2.2.3 Parallelized Preprocessor

HyperSpec’s parallelized preprocessor reduces the preprocessing time by vectorizing the computation. Specifically, the preprocessing operations are parallelized to multiple CPU cores using just-in-time (JIT) compilation by Numba.^15^ The JIT compilation requires negligible human intervention while providing great portability for code extension and modification.

These modules are used to preprocess the spectra as follows. First, peaks related to the precursor ion or with <1% intensity than the base peak intensity are removed. Second, spectra with <5 valid peaks or with a <250 Da mass range between their minimum and maximum peaks are removed. Third, at most, 50 peaks with the highest intensities are retained, and the peak intensities are normalized to [0, 1] using their L2 norm.

### 2.3 Bucket Division

An important challenge while clustering large data sets, with *n* spectra, is that performing all pairwise spectrum comparisons results in a dense pairwise distance matrix with quadratic  complexity, which is prohibitive for large *n*. To reduce this requirement, we follow a simple and effective strategy by dividing spectra into buckets.^7,9^ After all MGF files have been processed by the spectrum preprocessing module, the spectra are sorted and organized by ascending precursor *m*/*z* order (Figure 1b). Instead of having to cluster an entire data set, the spectra are divided into smaller buckets as follows
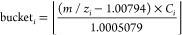
1where *C*_*i*_ is the precursor charge, *m*/*z*_*i*_ is the precursor *m*/*z* associated with the *i*th spectrum, 1.00794 is the mass of the charge, and 1.0005079 corresponds to the distance between the centers of two adjacent clusters of physically possible peptide masses.^16^ Each bucket is represented using an integer.

This bucket division scheme significantly reduces the memory usage and runtime by only comparing spectra within the same bucket to compute distance matrices for each bucket, instead of having to perform all pairwise spectrum comparisons for the full data set.

### 2.4 GPU-Accelerated Spectral Clustering in Hyperdimensional Space

HyperSpec exploits emerging HDC techniques^13,17^ to convert the preprocessed spectra into hyperdimensional space, where data are expressed as high-dimensional vectors with binary values. An important advantage of such HDC encoding is that the transformed data preserve features of the original space while exhibiting opportunities for data parallelism that can be leveraged by parallel GPU architectures.^17^ Due to this reason, the final three steps of HyperSpec (HD encoding, HD distance computation, and clustering) can be significantly accelerated using a GPU or CPU. HyperSpec clusters spectra by bucket granularity, meaning that one bucket is encoded, computed, and clustered at a time.

#### 2.4.1 HD Encoding for Spectra

**Figure 3:**
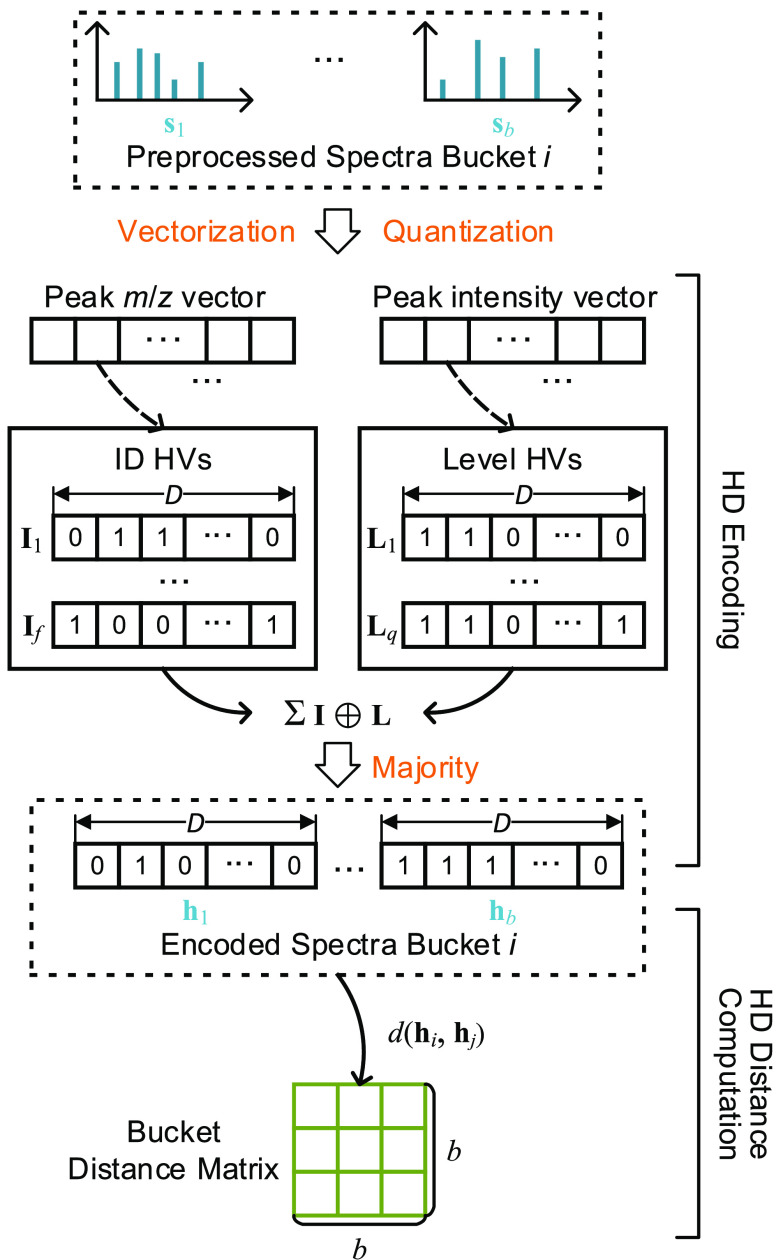
HD encoding and distance computation on a GPU. Each preprocessed spectrum’s *m*/*z* and intensity after vectorization and quantization are encoded into single hypervector (HV). Then, the bucket distance matrix is computed.

Whereas previous works^4,6^ directly computed spectrum similarities and performed clustering using the peak vectors, HyperSpec first uses HD encoding to project spectra to binary hypervectors (HVs) in the hyperdimensional space before performing the distance calculations (Figure 3). The HD encoding models the locality of the peak *m*/*z* and intensity values using two sets of encoding HVs (ID HVs **I** and level HVs **L**, respectively). The ID HVs **I** ∈{ **I**_1_, **I**_2_, ..., **I**_*f*_} reflect the spatial locality of *m*/*z* values, while the level HVs **L**∈{ **L**_1_, **L**_2_, ..., **L**_*Q*_ } reflect the intensity of peaks, where *f* and *Q* are the maximum peak index range and intensity quantization levels, respectively. Both **I**_*i*_ and **L**_*j*_ are *D*-dimensional binary HVs, such that **I**_*i*_, **L**_*j*_ ∈{0,1}^*D*^.

The two sets of encoding HVs, **I** and **L**, are iteratively generated in a stochastic manner. For ID HVs **I**, first a random HV is generated and regarded as **I**_1_. Next, the *i*th HV **I**_*i*_ is generated by randomly flipping a specific number of bits from its preceding HV **I**_*i*–1_. In this work, the default number of flipped bits is . For level HVs **L**, the generation process of first HV **L**_1_ is identical with **I**. The difference is that level HVs generate the *i*th HV **L**_*i*_ by flipping  bits compared to the preceding HV **L**_*i*–1_. The impact of the generation parameters on the clustering quality is discussed in section 3.

In the HD spectrum encoding process, the spectra in each bucket are first converted and quantized to two sparse vectors: peak *m*/*z* and peak intensity vectors. Based on the *m*/*z* and intensity pairs (*i*, *j*) in the peak *m*/*z* and peak intensity vectors, HyperSpec’s HD encoder finds the associated ID HV *I*_*i*_ and level HV *L*_*j*_ in the encoding HV sets. The fetched ID HV *I*_*i*_ and level HV *L*_*j*_ are then pointwise XORed by *I*_*i*_ ⊕*L*_*j*_. After all , where  denotes the set of peak *m*/*z* and intensity vectors, are computed, the HD-encoded spectrum is generated as follows
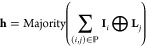
2where Majority(·) denotes the pointwise majority function that generates the binarized spectrum HV **h**∈{0, 1}^*D*^.

The HD dimension *D* needs to be large (normally > 1000) to guarantee representation capability.^17^ However, because such a large dimension incurs an expensive encoding cost, we have made two optimizations to reduce the encoding overhead.

#### 2.4.2 Bit Packing

By default, existing CPU or GPU architectures have a byte-level data granularity. However, storing a binary HV as a byte array needs 8× larger space than the theoretical number of bits *D*. To increase the memory efficiency of HyperSpec, HVs are stored in a bit-packed data structure, where every 32 bits of a HV **h** are packed into a 32-bit integer and each HV is stored in an integer array with length , which reduces the memory requirements to store a HV 8-fold.

#### 2.4.3 Batched GPU Parallel Encoding

The HD encoding is a bit-parallel algorithm, such that each bit of **h** can be computed independently. We have implemented the HD encoding modules using the CUDA platform^18^ and the HDC-specialized GPU memory optimization scheme^19^ to exploit this parallelism on GPUs. Before starting the HD encoding process, ID HVs **I** and level HVs **L** are transferred to the GPU memory. We found that data transfer of the HVs incurs a large overhead, since the size of the ID HVs **I** is much larger than the size of a single encoded spectrum. To reduce this overhead, the GPU parallel encoding in HyperSpec is performed in a batchwise manner, where the HV data are transferred while a batch of spectra are processed.

#### 2.4.4 HD Distance Computation

The clustering step of HyperSpec operates on the pairwise distance matrix of each bucket (also called bucket distance matrix). We use a normalized Hamming distance to measure the similarity between spectrum HVs. For two binary encoded spectra **h**_*i*_ and **h**_*j*_, the Hamming distance is first computed by counting the set bits of their XOR result **h**_*i*_⊕**h**_*j*_. Then the Hamming distance is normalized to [0, 1] by dividing *D*. Consequently, the pairwise distance *d*(**h**_*i*_, **h**_*j*_) is computed as
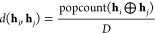
3where popcount(·) denotes the operation that obtains the number of set bits in a binary vector.

The HDC-based distance computation is lightweight because the computation of eq 3 only needs XOR and ones-counting operations. Our efficient implementation of these distance calculations leverages two CUDA integer intrinsics, XOR and popc. Additionally, by operating on bit-packed HVs, the time complexity to calculate each value in the pairwise distance matrix is reduced from *D* to .

#### 2.4.5 Clustering Algorithms

HyperSpec supports two popular clustering algorithms—DBSCAN^20^ and hierarchical clustering^21^—to cluster each spectra bucket based on the bucket distance matrix. HyperSpec implements these two clustering algorithms due to their three common benefits.

(1) DBSCAN and hierarchical clustering have been previously demonstrated effective to generate satisfactory quality for spectral clustering.^9,22,23^ The analysis in section 3.1 shows DBSCAN and hierarchical clustering yield various trade-offs between runtime and clustering quality. Supporting both of them allows the users to have more flexible choices.
(2) DBSCAN and hierarchical clustering require minimal efforts to tune the algorithmic hyperparameters, as the number of clusters does not need to be specified explicitly.
(3) From the perspective of runtime performance, the off-the-shelf fast DBSCAN and hierarchical clustering implementations^21,24^ are available and the clustering speed scales well to million- or even billion-scale scenarios.

### 2.5 Software Development and Code Availability

HyperSpec was implemented in Python 3.8. The MGF loading and parsing functions were written in C++ and compiled to Cython interfaces^25^ that can be invoked by Python. The spectrum preprocessing functionality was parallelized using the JIT compilation library Numba (version 1.20.2).^15^ The HD encoding and distance computation functions on a GPU were implemented using Numba and CuPy. We used the DBSCAN available in the cuML library (version 22.04)^24^ of the RAPIDS^26^ framework and fast hierarchical clustering with complete linkage in fastcluster^21^ to perform clustering on a GPU and CPU, respectively. HyperSpec is publicly available as open source at https://github.com/wh-xu/Hyper-Spec under the BSD license.

### 2.6 Clustering Evaluation

#### 2.6.1 Clustering Quality Metrics

We used the following metrics to evaluate the clustering quality and runtime performance.

- **Clustered spectra ratio.** The clustered spectra ratio equals the number of clustered spectra divided by the total number of spectra. This metric evaluates the clustering capability of the corresponding clustering tool.
- **Incorrect clustering ratio.** Incorrectly clustered spectra are those spectra whose peptide labels deviate from the most frequent peptide label in their clusters. The incorrect clustering ratio is the number of incorrectly clustered spectra divided by the total number of clustered and identified spectra.
- **Completeness.** Completeness measures the fragmentation of spectra corresponding to the same peptide across multiple clusters and is based on the notion of entropy in information theory. A clustering result that perfectly satisfies the completeness criterium (value “1”) assigns all PSMs with an identical peptide label to a single cluster. Completeness is computed as one minus the conditional entropy of the cluster distribution given the peptide assignments divided by the maximum reduction in entropy the peptide assignments could provide.^27^
- **Runtime.** The runtime is defined as the wall clock time between the start of spectrum preprocessing and the finish of the clustering procedure. We use the Linux system command to measure the wall clock time. The time for generating cluster consensus spectra was excluded since the overhead hereof is small.

#### 2.6.2 Hardware Configurations

The runtime performance of all clustering libraries was measured on a server with a 12-core CPU, 128 GB DDR4 memory, and a 2 TB NVMe solid-state drive (SSD). The equipped GPU card was an NVIDIA GeForce RTX 3090 GPU with 24 GB RAM. All tools were allowed to use all available processor cores and threads.

#### 2.6.3 Benchmarks

We compared HyperSpec to six state-of-the-art spectral clustering libraries, including GLEAMS,^23^ falcon,^9^ msCRUSH,^4^ MaRaCluster,^6^ spectra-cluster,^8^ and MS-Cluster.^5^ The clustering quality was controlled by varying the spectrum similarity threshold values, while the other configurations were set to the default values without explicit specifications. The distance threshold during clustering in HyperSpec was from 0.2 to 0.45. GLEAMS’ distance threshold for agglomerative clustering with complete linkage was 0.2–0.7. The cosine distance threshold of falcon was 0.05–0.25. msCRUSH’s cosine similarity threshold was varied from 0.3 to 0.8. MaRaCluster’s *P* value was from −30 to −3. The clustering threshold for spectra-cluster was 0.8–0.99999. MS-Cluster’s mixture probability was from 0.00001 to 0.1.

#### 2.6.4 Data Set

We used five MS data sets at different scales for evaluation (Table 1). These data sets consist of various human proteomics data, such as the HEK293 cell line,^2,28,29^ HeLa,^3^ and a draft map of the human proteome.^1^ For all data sets, raw files were downloaded from PRIDE^30^ and converted to MGF files using ThermoRawFileParser.^31^

**Table 1:** Properties of the Evaluated MS Data Sets

data set	sample type	PRIDE ID	no. of spectra	size
Data set-A	kidney cell^2^	PXD001468	1.1M	5.6 GB
Data set-B	kidney cell^28^	PXD001197	1.1M	25 GB
Data set-C	HeLa proteins^3^	PXD003258	4.1M	54 GB
Data set-D	HEK293 cell ^29^	PXD001511	4.2M	87 GB
Data set-E	human proteome draft ^1^	PXD000561	21.1M	131 GB

For each data set, spectra with precursor charge 2 and precursor charge 3 were considered. The largest data set, PXD000561,^1^ was used for runtime and clustering quality evaluation. The corresponding spectrum identifications were downloaded from MassIVE reanalysis RMSV000000091.3. These identifications were obtained via automatic reanalysis of public data on MassIVE using MS-GF+.^32^ Spectra were searched against the UniProtKB/Swiss-Prot human reference proteome (downloaded 2016/05/23)^33^ augmented with common contaminants. Search settings included a 50 ppm precursor mass tolerance, trypsin cleavage with maximum one nonenzymatic peptide terminus, and cysteine carbamidomethylation as a static modification. Methionine oxidation, formation of pyroglutamate from N-terminal glutamine, N-terminal carbamylation, N-terminal acetylation, and deamidation of asparagine and glutamine were specified as variable modifications, with a maximum one modification per peptide. The remaining four data sets were used for runtime evaluation only.

## 3 Results

### 3.1 Clustering Quality Comparison

#### 3.1.1 HyperSpec Clustering Quality Using Different Parameters

We studied the impact of HD parameters and clustering algorithms on HyperSpec’s clustering quality to select the optimal parameter combination. For HD, the two hyperparameters that influence the capability to represent spectra as HVs, and thus impact the spectrum clustering quality, are the HV dimension *D* and quantization level *Q*. Using the draft human proteome Data set-E, we examined the clustering quality using different combinations of clustering algorithms (DBSCAN or hierarchical clustering with complete linkage), HV dimension *D* (Supplementary Information Table S1), and quantization level *Q* (Supplementary Information Table S2), fixing the clustering distance threshold.

First, the HV dimension *D* was varied between 128 and 8192 and three clustering quality metrics (clustered spectra ratio, incorrect clustering ratio, and completeness) were computed for each combination of clustering algorithm and *D* value (Supplementary Information Table S1). This evaluation showed that as the HV dimension *D* increases, the incorrect clustering ratio and the clustered spectra ratio for both clustering algorithms decreased. However, the completeness of DBSCAN decreases from 0.8979 to 0.8615, while hierarchical clustering’s completeness is improved from 0.8071 to 0.8406. This is because a larger *D* allows the HVs to more granularly represent the spectra after encoding; their corresponding similarities will more accurately reflect the true spectral similarities and avoid that spectra corresponding to different peptides are incorrectly grouped together. The clustering results become less complete for DBSCAN as the density-based DBSCAN is unable to form large clusters when the spectral similarities are more accurate. Larger *D* also increases the memory usage for HV encoding and storing. The HV dimension *D* = 2048 balances well between clustering quality and memory consumption. We used *D* = 2048 as the default value for HV dimension.

In the second experiment, we fixed *D* = 2048 and then varied the quantization level *Q* from 4 to 64 and calculated the corresponding clustering quality metrics for each quantization level (Supplementary Information Table S2). Increasing quantization level *Q* reduced the clustered spectra ratio as well as completeness while slightly improving the incorrect clustering ratio for both clustering algorithms. For DBSCAN, the incorrect clustering ratio dropped from 1.41% to 1.29% while completeness dropped from 0.8644 to 0.8595 as *Q* is increasing from 4 to 64. Overall, the clustering quality is less sensitive to the change of quantization level *Q*. We choose *Q* = 16 as the default value for quantization level.

We find hierarchical clustering with complete linkage achieves a better clustering spectra ratio and lower incorrect clustering ratio as compared to DBSCAN (see Supplementary Information Tables S1 and S2). In the following sections, we use hierarchical clustering as the default clustering algorithm without explicit specifications.

#### 3.1.2 Comparison with Existing Tools

**Figure 4:**
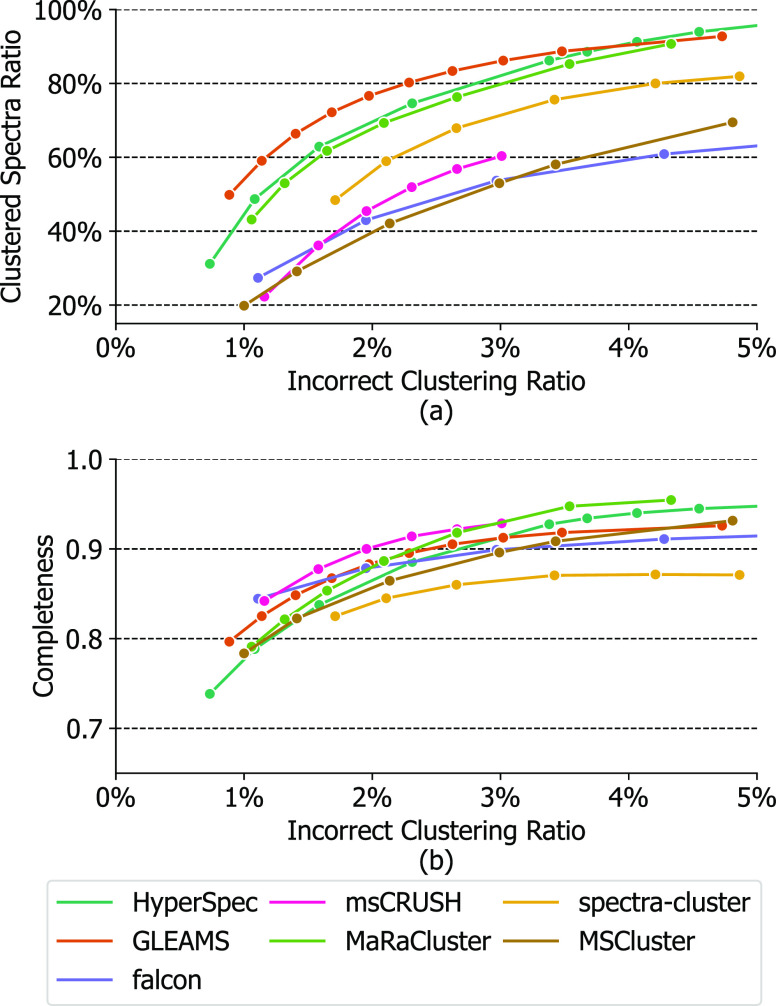
Clustering quality comparison for seven clustering tools: (a) clustered spectra ratio vs incorrect clustering ratio, (b) clustering completeness vs incorrect clustering ratio.

Using the draft human proteome Data set-E, we also compared the clustering quality of HyperSpec to six alternative spectral clustering tools (Figure 4). As suggested previously,^8,22^ a high clustered spectra ratio at a low incorrect clustering ratio indicates a better clustering capability for a specific tool. Additionally, completeness measures fragmentation of the same peptide over multiple clusters, and an ideal clustering result should be as complete as possible to ensure that spectra originating from the same peptide are more likely to be grouped into a single cluster.

For the clustered spectra ratio shown in Figure 4a, HyperSpec is significantly higher than falcon and MS-Cluster across different incorrect clustering ratios. Meanwhile, HyperSpec consistently clusters more spectra than MaRaCluster and is slightly inferior to GLEAMS, achieving the second best result at the region with low incorrect clustering ratio.

In terms of completeness, HyperSpec outperforms spectra-cluster, MS-Cluster, and falcon, achieving top-3 completeness among the six clustering tools according to Figure 4b. In contrast to falcon and spectra-cluster, which reach a plateau in terms of completeness as the incorrect clustering ratio increases, HyperSpec is able to trade off a small amount of incorrect clustering ratio for more complete clustering results. HyperSpec also maintains high completeness values as the incorrect clustering ratio increases. For the region with incorrect clustering ratio > 3%, HyperSpec surpasses other counterparts except for MaRaCluster, suggesting that the clusters produced by HyperSpec are generally less fragmented. This can be especially beneficial for downstream analysis tasks since more complete clustering results can be represented by a smaller number of consensus spectra to optimally minimize data redundancy.

**Figure 5:**
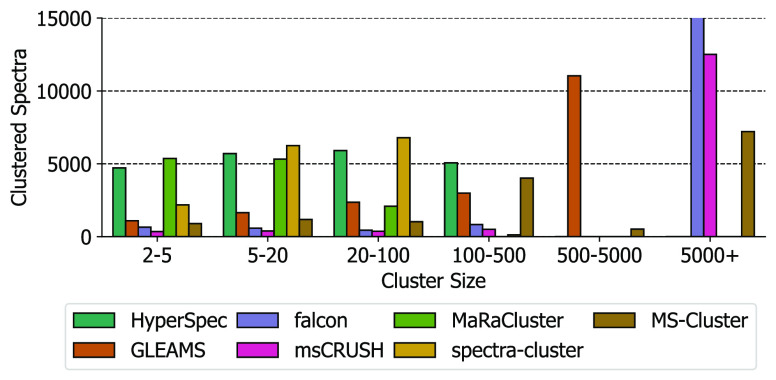
Distribution of cluster sizes for the most frequently identified peptide sequence VATVSIPR with precursor charge 2.

To intuitively understand the clustering results, we studied the distribution of cluster sizes for the most frequently identified peptide sequence VATVSIPR with precursor charge 2 for different spectral clustering tools (Figure 5). Here, HyperSpec used a threshold of *eps* = 0.25, HD dimension of *D* = 2048, and quantization level of *Q* = 16 to achieve a clustering with a ratio of incorrectly clustered spectra < 1.2%. The other spectral clustering tools use configurations as listed in Table 2. We can see that HyperSpec mostly forms medium-size clusters with size 5–500 as compared to falcon and msCRUSH which tend to generate large clusters (size > 500). The majority of clusters produced by MaRaCluster and spectra-cluster contain less than 100 spectra, which indicates that these two tools are more likely to group spectra corresponding to the same peptide into multiple small and fragmented clusters. The characteristics of cluster size distribution for HyperSpec is most similar to those of MaRaCluster and spectra-cluster that also adopt hierarchical clustering. In comparison, falcon and msCRUSH group these spectra into a limited number of large clusters that contain at least 5000 spectra. We also add the six most frequent peptide sequences on Data set-E with charge 2 and charge 3 as shown in (Supplementary Information Figure S2) to illustrate the distribution of the cluster sizes.

**Table 2:** Key Performance Metrics of HyperSpec, GLEAMS, falcon, msCRUSH, and MaRaCluster on the draft Human Proteome Data Set-E.

tool	parameters	runtime	peak memory	clustered spectra	incorrect clustering ratio	completeness
HyperSpec	*eps* = 0.25, *D* = 2048, *Q* = 16	24 min	54 GB	10 290 245 (48.70%)	1.08%	0.7885
GLEAMS	threshold = 0.25	217 min	34 GB	12 392 427 (59.06%)	1.14%	0.8251
falcon	*eps* = 0.05	161 min	87 GB	5 675 468 (27.42%)	1.11%	0.8438
msCRUSH	similarity = 0.8	55 min	91 GB	4 397 921 (22.34%)	1.16%	0.8418
MaRaCluster	pvalThreshold = −30.0	251 min	19 GB	9 305 471 (43.20%)	1.07%	0.7911

### 3.2 Spectra Database Searching Comparison

The generated consensuses from spectra clustering tools can be used for the downstream spectra database search to identify peptide sequences. We compared the spectra searching performance on the human proteome draft Data set-E in Table 1 for three clustering tools, including HyperSpec, GLEAMS, and falcon. The clustering results generated by these tools were controlled to have a clustered spectra ratio around 60%. The clustering consensuses of HyperSpec were generated based on the original raw spectra data. We use the default parameters provided by the software except for the distance thresholds. Specifically, HyperSpec uses a distance threshold value = 0.3 and produces 62.9% clustered ratio with 1.58% incorrect clustering ratio. GLEAMS uses a distance threshold value = 0.25 and produces 59.1% clustered ratio with 1.14% incorrect clustering ratio. falcon uses a distance threshold value = 0.2 and produces 61.1% clustered ratio with 4.27% incorrect clustering ratio. The clustering consensuses were searched using MSGF+^32^ with the same parameters given in section 2.6.4.

**Figure 6:**
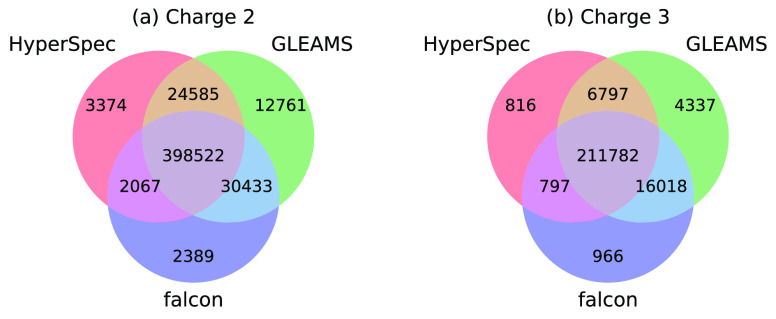
Venn diagrams that depict the overlap of identified unique peptides using consensus spectra generated by HyperSpec, GLEAMS, and falcon. The precursor charges include charge 2 in a and charge 3 in b. Identified peptides from HyperSpec are highly overlapped with the results generated by GLEAMS and falcon.

Figure 6 illustrates the Venn diagrams that depict the overlap relationship of identified unique peptides using consensus spectra clustered by HyperSpec, GLEAMS, and falcon. GLEAMS identifies the largest number of unique peptides. HyperSpec identifies 8.1% and 1.1% less unique peptides for charge 2 and identifies 7.8% and 4.1% less unique peptides for charge 3 as compared to GLEAMS and falcon, respectively. It should be noted that HyperSpec achieves a much lower incorrect clustering ratio than falcon (1.58% vs 4.27%). Considering that HyperSpec is significantly faster than GLEAMS and falcon, we believe its slight degradation of spectra searching quality is acceptable. Furthermore, HyperSpec not only boosts the spectra clustering procedure but also reduces the search time of spectra database search. HyperSpec yields about a 2.7× speedup over the spectra searching using raw spectra because the redundant searching processes for those similar spectra are skipped.

### 3.3 Runtime Performance Comparison

Runtime is a crucial metric to evaluate the efficiency of spectral clustering tools. Especially, to be able to perform spectral clustering at the repository scale, tools have to be fast to handle the ever-growing amount of MS data that is available in public data resources.

We first compared the total clustering time of HyperSpec using DBSCAN or hierarchical clustering with complete linkage on five data sets. Supplementary Information Figure S3 shows that hierarchical clustering was ∼29% faster than DBSCAN on the small-size and medium-size Data set-A to Data set-D. However, HyperSpec using DBSCAN generated more complete results with 38% shorter runtime than hierarchical clustering on large-scale data set Data set-E. The shorter runtime on large-scale data sets comes from the optimized DBSCAN routines on parallel GPU devices.

**Figure 7:**
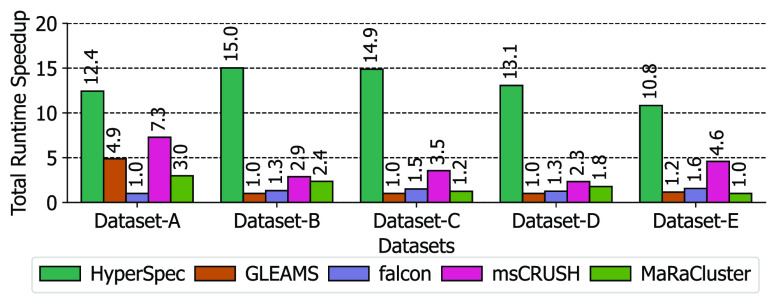
Total clustering runtime speedup of HyperSpec compared to alternative clustering tools. The tool with the slowest runtime on each data set was normalized to 1.

**Figure 8:**
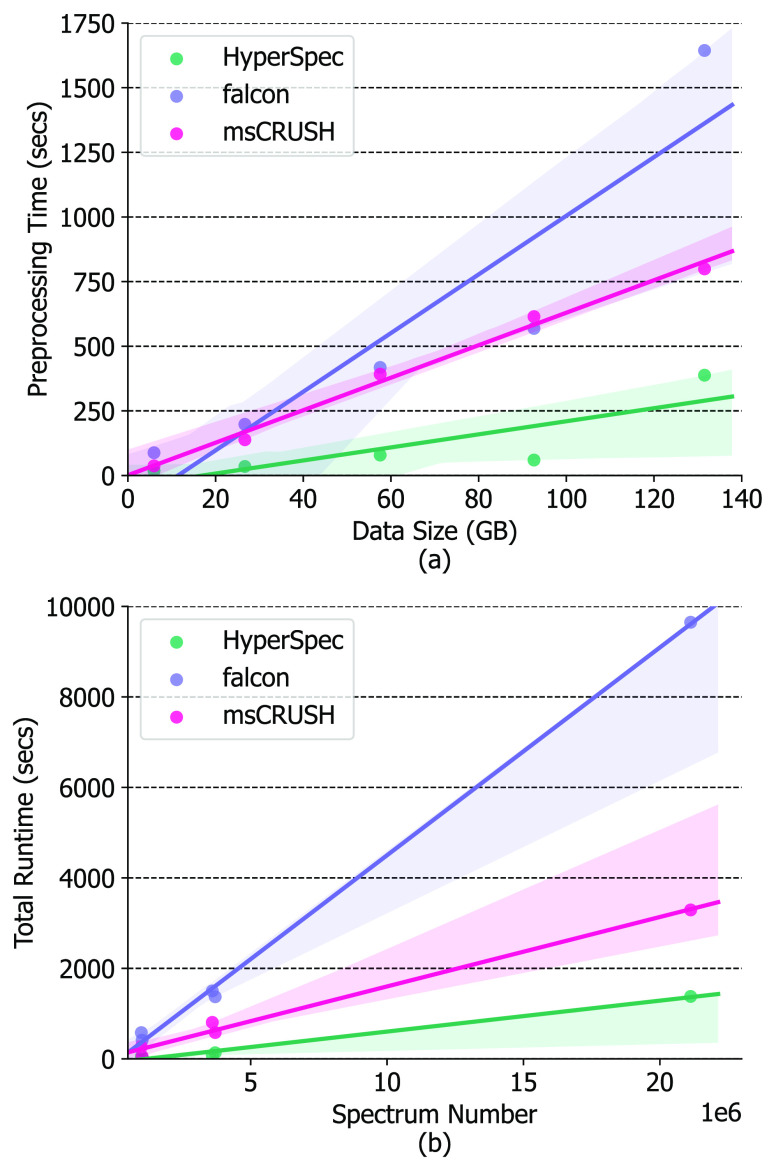
Runtime performance of msCRUSH,^4^ falcon,^9^ and HyperSpec when scaling to different data set sizes and number of spectra.

We extensively measured the runtime performance of HyperSpec hierarchical clustering with complete linkage compared to three fast spectral clustering tools on five data sets with a varying number of spectra in Table 1. falcon and GLEAMS are Python-based libraries that use both optimized JIT compilation and multiprocessing, while msCRUSH and MaRaCluster were written in high-performance C++ and optimized using multithreading. spectra-cluster and MS-Cluster were not considered here since they are significantly (>5×) slower than other tools. Our evaluation results in Figure 7 indicate that HyperSpec consistently outperforms all other tools in terms of runtime; 10.8 × to 15.0 × speedup was observed across different data sets. HyperSpec’s speed advantage for spectra preprocessing progressively grows for larger data sets (Figure 8a). We further investigated the runtime scalability when processing an increasing number of spectra (Figure 8b). Our analysis shows HyperSpec’s excellent scalability and performance advantages over alternative tools for increasingly large MS data sets.

We also studied detailed performance metrics (runtime, peak memory consumption, and clustering quality) when running HyperSpec, GLEAMS, falcon, msCRUSH, and MaRaCluster on the draft human proteome Data set-E (Table 2). All tools were allowed to use all available CPU cores to obtain the fastest clustering speed and were configured to produce a clustering result with a ratio of incorrect clustered spectra around 1.0%. HyperSpec was able to process the full draft human proteome data set, amounting to 131 GB of MS data, in a mere 24 min, which is by far the fastest speed among the four spectral clustering tools considered. This runtime is 2.3× faster than the second-fastest tool, msCRUSH, while achieving a higher clustered spectra ratio and smaller peak memory usage. Although GLEAMS produced the highest ratio of correctly clustered spectra, it required 217 min of processing time, which is 9.0× slower than HyperSpec. This is because >90% of GLEAMS’ runtime is consumed by the spectra preprocessing and embedding steps. MaRaCluster obtained the lowest ratio of incorrectly clustered spectra among all tools and a comparable completeness value with HyperSpec. Finally, with a peak memory consumption of 54 GB, HyperSpec was more memory efficient than msCRUSH and falcon. In summary, because HyperSpec achieves an optimal tradeoff between clustering quality and runtime efficiency, it is an especially appealing option to process the quickly growing volumes of MS data.

## 4 Discussion

Here, we have presented a HDC-based spectral clustering tool, HyperSpec, to achieve both excellent clustering quality and runtime. Instead of clustering raw spectra directly, HyperSpec leverages HDC^17^ to convert spectra to hyperdimensional space. Specifically, the spectra are first encoded into binary HVs that have high dimensionality but simpler representation format. Our evaluations show that HyperSpec achieves a comparable clustering quality as state-of-the-art spectral clustering tools.^4−6,9,34^ Furthermore, we profiled and analyzed the bottlenecks of existing clustering tools. We developed optimized spectra preprocessing routines and an efficient clustering flow by addressing bottleneck components. As a result, HyperSpec achieved the fastest speed among all tools considered and is orders of magnitude faster than alternative spectral clustering tools.

HyperSpec is extensible to plug in and support other MS workloads. For example, spectrum preprocessing is a common step during various MS data analysis tasks, such as sequence database searching^35^ and spectral library searching.^34,36^ The spectrum preprocessing routines in HyperSpec are highly modularized, so that users can easily integrate these optimized routines into other workloads to take advantage of their efficient implementations.

Another potential application of HyperSpec is to utilize the compact binary HV representation to compress MS data. We have demonstrated that the original spectra in floating-point format can be encoded into binary HVs with *D* = 1024–4096 bits with minimal loss of information to maintain a high-quality clustering quality. In this case, the original spectrum with 50–100 peaks in 32-bit or 64-bit floating-point number can be compressed by a factor of 3.1–12.5×. Moreover, HV encoding could be convenient for the subsequent downstream MS workloads, such as spectrum identification. Specifically, off-the-shelf HDC-based pattern matching algorithms^37−39^ could be leveraged to match spectra against a peptide database.

There are still several opportunities to improve upon HyperSpec’s clustering quality and runtime performance. Similar to MaRaCluster and spectra-cluster,^6,34^ one possible approach could be to derive an optimized distance function to compare spectrum HVs and improve the clustering quality, since finding similar spectra is an essential task during spectral clustering. Another strategy could be to adopt a postprocessing scheme after clustering to split up invalid clusters.^7,22^ To further shorten the clustering runtime, the HV distance computations and the clustering step can be parallelized over multiple GPU cards. Because the bucket division mechanism relaxes data dependencies between different buckets of spectra and the clustering implementations in cuML^24^ natively support multiple GPUs, a multi-GPU mode could be integrated in HyperSpec at minimal effort to achieve a near-linear speedup. The other possible speedup opportunity is combining HyperSpec with the emerging near-storage spectrum processing hardware^40^ that can generate higher energy efficiency for repository-scale data processing.

## Supporting Information Available

Clustering quality on Data set-E for different clustering algorithms and values of HD dimension *D.*; clustering quality on Data set-E for different clustering algorithms and values of HD quantization level *Q*; runtime of multiprocessing-based preprocessing using different CPU cores on Data set-E; distribution of cluster sizes for the six most frequently identified peptide sequences on Data set-E with precursor charge 2 and charge 3; runtime comparison for HyperSpec with DBSCAN and hierarchical clustering with complete linkage on five data sets (PDF)
