# Supplementary material for: HyperSpec: Ultrafast Mass Spectra Clustering in Hyperdimensional Space
Source: J Proteome Res. 2023 May 11;22(6):1639–48. doi: 10.1021/acs.jproteome.2c00612 (PMC10243109; doi:10.1021/acs.jproteome.2c00612)
Supplement: Supplementary file 1 — pr2c00612_si_001.pdf [file pr2c00612_si_001.pdf]

HyperSpec: Ultra-fast Mass Spectra Clustering in Hyperdimensional Space

Weihong Xu,*,[1] Jaeyoung Kang,[2] Wout Bittremieux,[3] Niema Moshiri,[1] and Tajana Rosing[1]

[1]*Department of Computer Science Engineering, University of California, San Diego, La Jolla, CA 92093, USA*

[2]*Department of Electrical and Computer Engineering, University of California, San Diego, La Jolla, CA 92093, USA*

[3]*Skaggs School of Pharmacy and Pharmaceutical Science, University of California, San Diego, La Jolla, CA 92093, USA*

E-mail: wexu@ucsd.edu

List of Supplementary Materials

- **Supplementary Table 1**

  Clustering quality on Dataset-E for different clustering algorithms and values of HD dimension D. DBSCAN and hierarchical clustering with complete linkage were used.

- **Supplementary Table 2**

  Clustering quality on Dataset-E for different clustering algorithms and values of HD quantization level Q. DBSCAN and hierarchical clustering with complete linkage were used.

- **Supplementary Figure 1**

  Runtime of multiprocessing-based preprocessing using different CPU cores on Dataset-E.

- **Supplementary Figure 2**

  Distribution of cluster sizes for the six most frequently identified peptide sequences on Dataset-E with precursor charge 2 and charge 3.

- **Supplementary Figure 3**

  The empirical cumulative distribution of the cluster sizes on Dataset-E for HyperSpec, GLEAMS, falcon, msCRUSH, and MaRaCluster.

- **Supplementary Figure 4**

  Runtime comparison for HyperSpec with DBSCAN and hierarchical clustering with complete linkage on five datasets. The default configurations were used for HyperSpec except for the clustering algorithm.

Supplementary Table 1: Clustering quality on Dataset-E for different clustering algorithms and values of HD dimension D. DBSCAN and hierarchical clustering with complete linkage were used. The default distance threshold was $eps = 0.2$ for DBSCAN and $eps = 0.3$ for hierarchical clustering with complete linkage, respectively. The HD quantization level was set to $Q = 16$. DBSCAN generally generated more complete results but less clustered spectra compared to hierarchical clustering. When increasing the HD dimension from $D = 128$ to $D = 4096$, the incorrect clustering ratios and clustering spectra ratios of both two clustering algorithms decrease. DBSCAN's completeness decreases while hierarchical with complete linkage's completeness improves. $D = 2048$ keeps a good balance between memory consumption and clustering quality.

**Algorithm**	**HD Dimension** D	**Quantization Level** Q	**Clustered Spectra**	**Incorrect Clustering Ratio**	**Completeness**
DBSCAN	$D = 128$	$Q = 16$	8 001 982 (37.89%)	2.17%	0.8979
DBSCAN	$D = 256$	$Q = 16$	7 107 355 (33.66%)	1.62%	0.8829
DBSCAN	$D = 512$	$Q = 16$	6 365 562 (30.14%)	1.40%	0.8693
DBSCAN	$D = 1024$	$Q = 16$	5 985 007 (28.34%)	1.34%	0.8656
DBSCAN	$D = 2048$	$Q = 16$	5 958 478 (28.20%)	1.30%	0.8638
DBSCAN	$D = 4096$	$Q = 16$	5 847 470 (27.67%)	1.28%	0.8615
Hierarchical	$D = 128$	$Q = 16$	14 809 404 (70.09%)	2.16%	0.8071
Hierarchical	$D = 256$	$Q = 16$	13 643 733 (64.57%)	1.67%	0.8154
Hierarchical	$D = 512$	$Q = 16$	13 266 246 (62.78%)	1.57%	0.8242
Hierarchical	$D = 1024$	$Q = 16$	13 301 599 (62.95%)	1.58%	0.8331
Hierarchical	$D = 2048$	$Q = 16$	13 289 980 (62.90%)	1.59%	0.8377
Hierarchical	$D = 4096$	$Q = 16$	13 292 059 (62.90%)	1.59%	0.8406

Supplementary Table 2: Clustering quality on Dataset-E for different clustering algorithms and values of HD quantization level Q. DBSCAN and hierarchical clustering with complete linkage were used. The default distance threshold was $eps = 0.2$ for DBSCAN and $eps = 0.3$ for hierarchical clustering with complete linkage, respectively. The quantization level has less impact on clustering quality compared to HD dimension. The HD dimension was set to $D = 2048$. DBSCAN generally generated more complete results but less clustered spectra compared to hierarchical clustering. When increasing the HD quantization level from $Q = 4$ to $Q = 64$, the three clustering quality metrics (incorrect clustering ratio, clustering spectra ratio, and completeness) of both clustering algorithms decrease. The quantization level $Q = 16$ was picked as the default value.

**Algorithm**	**HD Dimension D**	**Quantization Level Q**	**Clustered Spectra**	**Incorrect Clustering Ratio**	**Completeness**
DBSCAN	$D = 2048$	$Q = 4$	6 278 284 (29.73%)	1.41%	0.8644
DBSCAN	$D = 2048$	$Q = 8$	6 092 636 (28.85%)	1.33%	0.8621
DBSCAN	$D = 2048$	$Q = 16$	5 958 478 (28.20%)	1.30%	0.8638
DBSCAN	$D = 2048$	$Q = 32$	5 931 716 (28.09%)	1.29%	0.8596
DBSCAN	$D = 2048$	$Q = 64$	5 930 616 (28.08%)	1.29%	0.8595
Hierarchical	$D = 2048$	$Q = 4$	13 543 166 (64.10%)	1.66%	0.8415
Hierarchical	$D = 2048$	$Q = 8$	13 362 120 (63.24%)	1.60%	0.8389
Hierarchical	$D = 2048$	$Q = 16$	13 289 980 (62.90%)	1.59%	0.8378
Hierarchical	$D = 2048$	$Q = 32$	13 301 802 (62.95%)	1.59%	0.8377
Hierarchical	$D = 2048$	$Q = 64$	13 302 179 (62.96%)	1.59%	0.8377

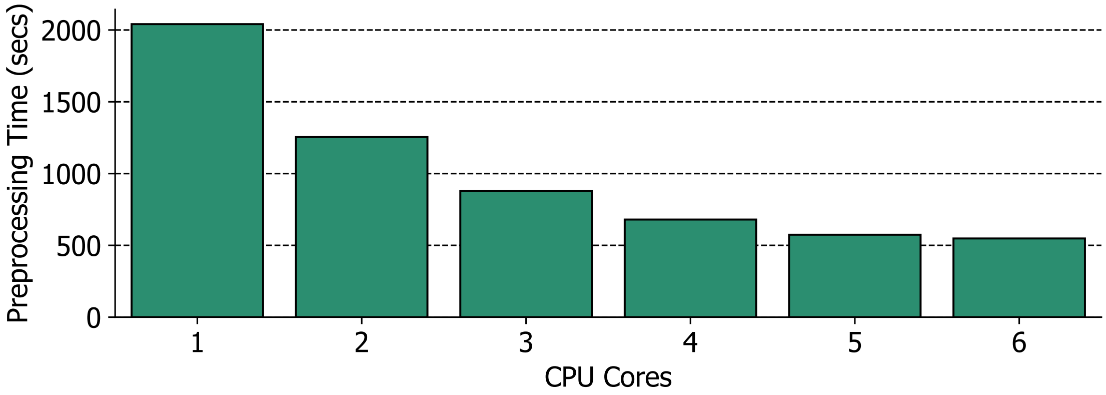

Supplementary Figure 1: Runtime of multiprocessing-based preprocessing using different CPU cores on Dataset-E. The CPU multiprocessing achieves sub-linear speedup for spectra preprocessing. When increasing the number of activated CPU cores from 1 to 6, the preprocessing runtime decreases from ≈ 2000 to ≈ 500. The performance gain of multiprocessing saturates when the number of activated CPU cores is more than 6.

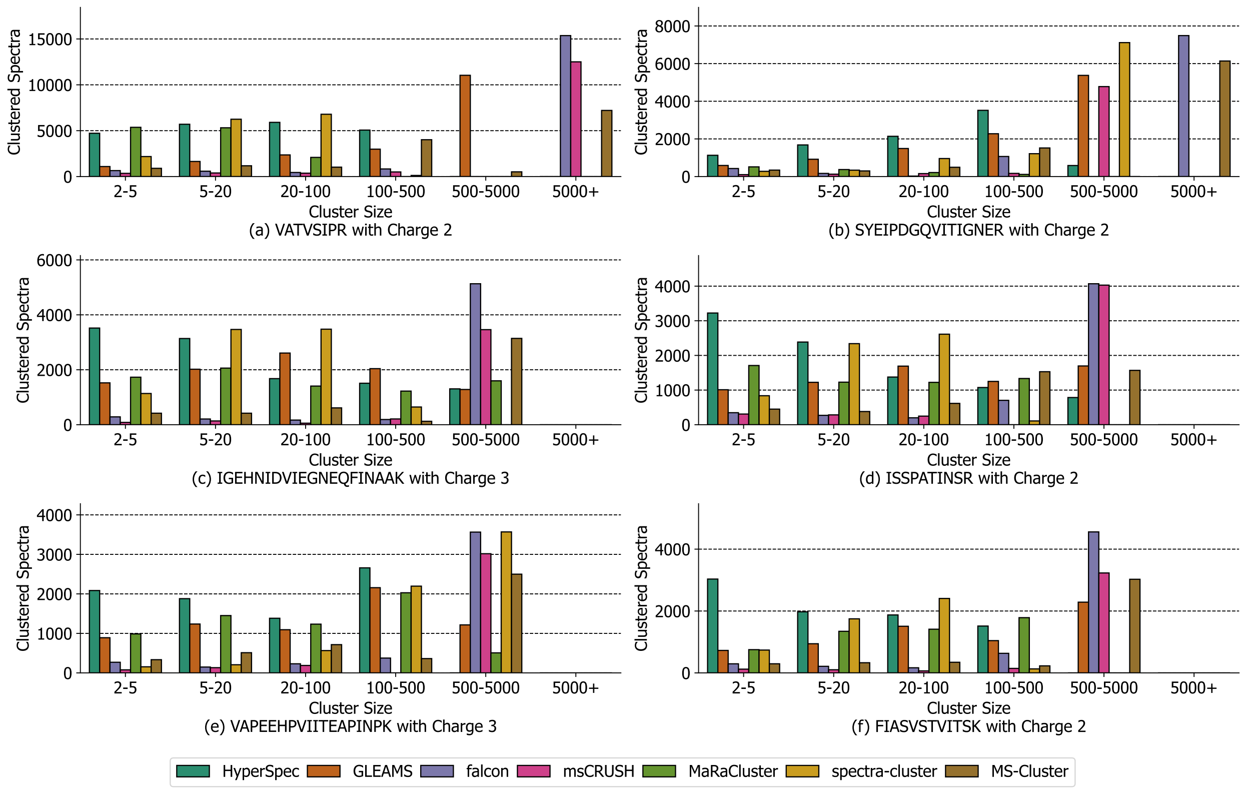

Supplementary Figure 2: Distribution of cluster sizes for the six most frequently identified peptide sequences on Dataset-E with precursor charge 2 and charge 3. Hierarchical clustering with complete linkage and distance threshold $ep = 0.25$ was used for HyperSpec. The incorrect clustering ratios for all clustering results were controlled at around 1.0%. HyperSpec tends to form medium-size clusters from size 5 to 500.

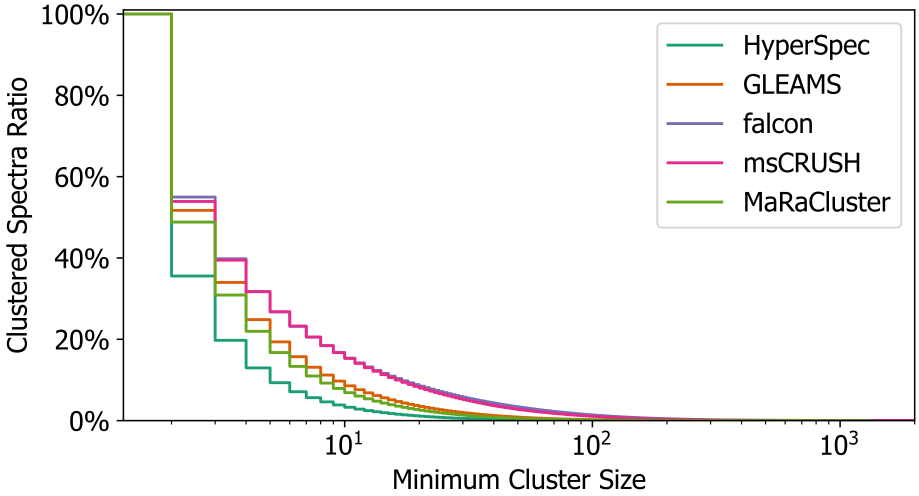

Supplementary Figure 3: The empirical cumulative distribution of the cluster sizes for HyperSpec, GLEAMS, falcon, msCRUSH, and MaRaCluster. The evaluated dataset is Dataset-E. Hierarchical clustering with complete linkage and distance threshold $ep = 0.25$ was used for HyperSpec. The incorrect clustering ratios for all clustering results were controlled at around 1.0%. The clusters in HyperSpec with size < 10 contribute to majority ($> 90\%$) of the clustered spectra. The increased number of clustered spectra is driven by the small cluster sizes.

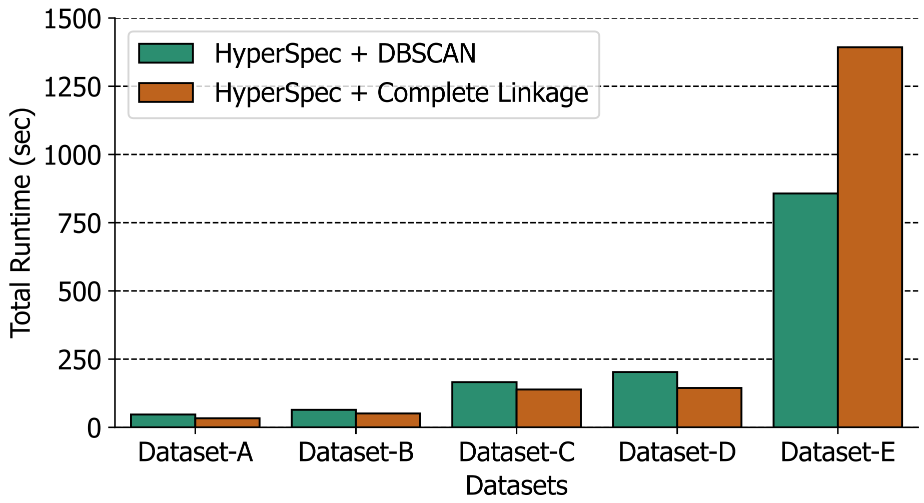

Supplementary Figure 4: Runtime comparison for HyperSpec with DBSCAN and hierarchical clustering with complete linkage on five datasets. The default configurations were used for HyperSpec except for the clustering algorithm. Hierarchical clustering was $\approx 29\%$ faster than hierarchical clustering with complete linkage on Dataset-A to Dataset-D. DBSCAN obtained 38% shorter runtime on the large-scale Dataset-E.
